# Brazilian research on cognitive impairment and dementia from 1999 to 2013

**DOI:** 10.1590/S1980-57642014DN84000015

**Published:** 2014

**Authors:** André Aguiar Souza Furtado de Toledo, Ricardo Nitrini, Cássio Machado de Campos Bottino, Paulo Caramelli

**Affiliations:** 1 Departamento de Clínica Médica, Faculdade de Medicina da Universidade Federal de Minas Gerais, Belo Horizonte, MG, Brasil.; 2 Departamento de Neurologia da Faculdade de Medicina da Universidade de São Paulo, SP, Brasil.; 3 Departamento de Psiquiatria da Faculdade de Medicina da Universidade de São Paulo, SP, Brasil.

**Keywords:** dementia, mild cognitive impairment, Alzheimer's disease, scientific output, Brazil, Brazilian institutions

## Abstract

The phenomenon of demographic transition in recent decades has increased the number of elderly people in Brazil, promoting an escalation in chronic-degenerative conditions, particularly dementia and cognitive related disorders.

**Objective:**

The aim of this study was to assess the evolution of the Brazilian scientific publications on dementia and related conditions from 1999 to 2013.

**Methods:**

Articles published during the analysis period were searched on three electronic databases: Scopus, Medline (via PubMed) and Lilacs (via BVS). The keywords used were Alzheimer's disease, dementia and mild cognitive impairment, with Brazil as the country of affiliation.

**Results:**

A total of 1,657 articles met the conditions for inclusion in the study. The output of Brazilian researchers in the area of cognitive disorders increased 11.38-fold in the fifteen-year period of analysis and 4.98-fold from 2003 to 2013. More than half of the articles (53%) were published in international journals. The majority of institutions involved in publications were public universities while 19% were collaborative studies involving Brazilian and international institutions.

**Conclusion:**

Despite marked growth, the number of Brazilian scientific publications in the area of cognitive impairment and dementia is still low. More effort is required to improve the output of Brazilian researchers and institutions. Possible strategies to accomplish this increase could be to encourage residents to participate in publications of scientific papers during their residence program and to increase the collaborations between different institutions within Brazil and with the international scientific community.

## Abstract (pt)

O fenômeno da transição demográfica nas últimas décadas aumentou o número de indivíduos idosos no Brasil, promovendo um avanço de condições crônico-degenerativas, particularmente demência e outros distúrbios cognitivos relacionados.

**Objetivo:**

A intenção deste estudo foi avaliar a evolução da produção científica brasileira em demência e condições relacionadas de 1999 a 2013.

**Métodos:**

Pesquisamos artigos publicados durante esse período em três bancos de dados eletrônicos: Scopus, Medline (via PubMed) e Lilacs (via BVS). As palavras-chave utilizadas foram doença de Alzheimer, demência e comprometimento cognitivo leve, com o Brasil como país de afiliação.

**Resultados:**

Um total de 1.657 artigos cumpriram as condições para inclusão no estudo. A produção de pesquisadores brasileiros na área de distúrbios cognitivos aumentou 11,38 vezes no período de análise e 4,98 vezes de 2003 a 2013. Mais da metade dos artigos (53%) foi publicada em periódicos internacionais. A maioria das instituições envolvidas em publicações eram universidades públicas e 19% eram estudos colaborativos envolvendo instituições brasileiras e internacionais.

**Conclusão:**

Apesar do crescimento expressivo, o número de publicações cientificas brasileiras na área de comprometimento cognitivo e demência é ainda baixo. Mais esforços são necessários para melhorar a produção de pesquisadores e instituições brasileiras. Possíveis estratégias para cumprir essa tarefa poderiam ser encorajar residentes a participar de publicações de artigos científicos durante seu programa de residência e aumentar as colaborações entre diferentes instituições brasileiras e com a comunidade científica internacional.

## INTRODUCTION

The phenomenon of demographic transition occurring worldwide, including in Brazil over recent decades, has produced a significant increase in the number of elderly people in the Brazilian population. An important consequence of this process is an increase in chronic-degenerative conditions, particularly dementia and cognitive related disorders. The most common causes of dementia are Alzheimer's disease, vascular dementia, frontotemporal dementia and Lewy body dementia.^1,2^ Another major clinical condition is mild cognitive impairment (MCI), denoting a condition where cognition lies between normality and dementia and may be a precursor of dementia.^3^

According to a recent meta-analysis conducted in Brazil on the prevalence of dementia among elderly Brazilians, dementia was most prevalent among poor, illiterate, female and very elderly individuals, although overall prevalence could not be estimated due to wide variations in the Brazilian population throughout the country or due to methodological differences across the studies.^4^

Efforts to improve the quality and availability of care to patients affected by these conditions call for greater investment not only in the health system but also on research into pathology, genetics, clinical aspects, treatment and modifiable risk factors.^5^

Alzheimer's disease is the most prevalent neurodegenerative condition, representing the main cause of dementia worldwide and also in Latin America.^6,7^ Due to this epidemiological and also clinical relevance it is a major area of scientific research. Accordingly, publications on Alzheimer's disease represent around 17% of all neuroscience literature.^8^

The main objective of this study was to assess the extent and evolution of publications on cognitive impairment and dementia involving Brazilian institutions from 1999 to 2013 and to characterize this output considering regional and institutional distributions besides other aspects.

## METHODS

The publications included were related to all types of dementia and mild cognitive impairment. Articles published from 1999 to 2013 were retrieved from electronic databases, including Scopus, Medline (via PubMed) and Latin American and Caribbean Health Sciences Literature (Lilacs, via BVS). The keywords were dementia, Alzheimer's disease and mild cognitive impairment (Table 1). Results were filtered to yield publications involving at least one Brazilian institution by using country of affiliation criteria.

**Table 1:** Electronic search strategy.

Database	Search strategy
Scopus	KEY (“mild cognitive impairment” OR dementia OR “alzheimer disease”) AND PUBYEAR > 1998 AND PUBYEAR < 2014 AND (LIMIT-TO(AFFILCOUNTRY, “Brazil”))
Medline	(“mild cognitive impairment”[MeSH] OR dementia[MeSH] OR “alzheimer disease”[MeSH]) AND ((“1999/01/01”[PDAT] : “2013/12/31”[PDAT]) AND medline[sb] AND Brazil[Affiliation])
Lilacs	mh:(“mild cognitive impairment” OR dementia OR “alzheimer, disease”) AND (db:(“LILACS”) AND year_cluster:(“2013” OR “2012” OR “2011” OR “2010” OR “2009” OR “2008” OR “2007” OR “2006” OR “2005” OR “2004” OR “2003” OR “2002” OR “2001” OR “2000” OR “1999”) AND pais_afiliacao:(“^iBRAZIL^eBRASIL^pBRASIL”))

Each paper was classified according to the following criteria: research type, name of periodical, year, Brazilian institution, state and collaboration type. Papers were then compiled in an Excel file. Institutions and states of collaborative publications were defined by the first Brazilian author in order of appearance in the list of authorship. The impact factor (IF) of each periodical was obtained from the Journal Citation Reports^®^ (via ISI Web of Knowledge) and was also included in the Excel file. Periodicals not listed in the ISI Web of Knowledge had IF computed by SCImago Journal & Country Rank (Powered by Scopus) using the same formula created by Thomson Reuters. Studies were excluded if not related to dementia or cognitive impairment, not completely fulfilling the search criteria or not involving a Brazilian institution.

## RESULTS

**Figure 1:**
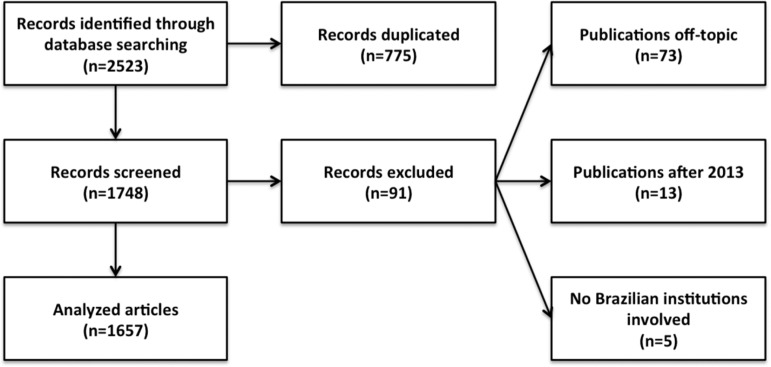
Flowchart of selection process.

A total of 2,523 articles were identified (Scopus: 1,449, Medline: 575, Lilacs: 499) of which 1,657 met the conditions for inclusion in the study (Figure 1).

**Figure 2:**
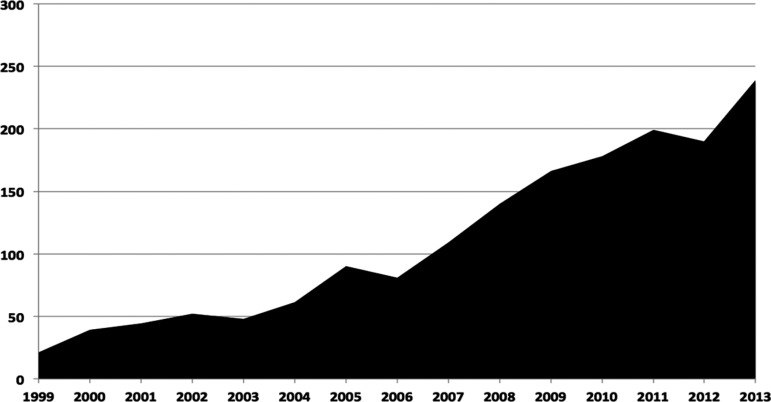
Evolution of publications over a fifteen-year period (1999-2013).

Considering the first and the last year of this study, the number of publications rose more than 1038% in fifteen years, with a marked increase since 2007 (Table 2, Figure 2).

**Table 2:** Publications by year (1999-2013).

Year	N	%
1999	21	1.27
2000	39	2.35
2001	44	2.66
2002	52	3.14
2003	48	2.90
2004	61	3.68
2005	90	5.43
2006	81	4.89
2007	109	6.58
2008	140	8.45
2009	166	10.02
2010	178	10.74
2011	199	12.01
2012	190	11.47
2013	239	14.42
Total	1657	100%

**Figure 3:**
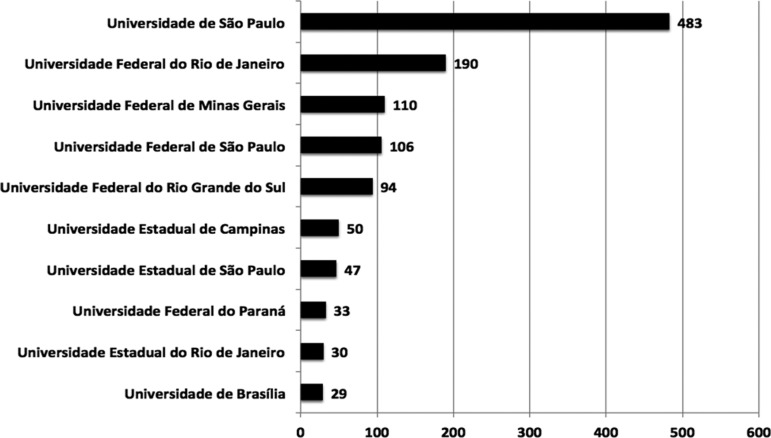
Number of publications according to Brazilian institutions (Top 10).

**Figure 4:**
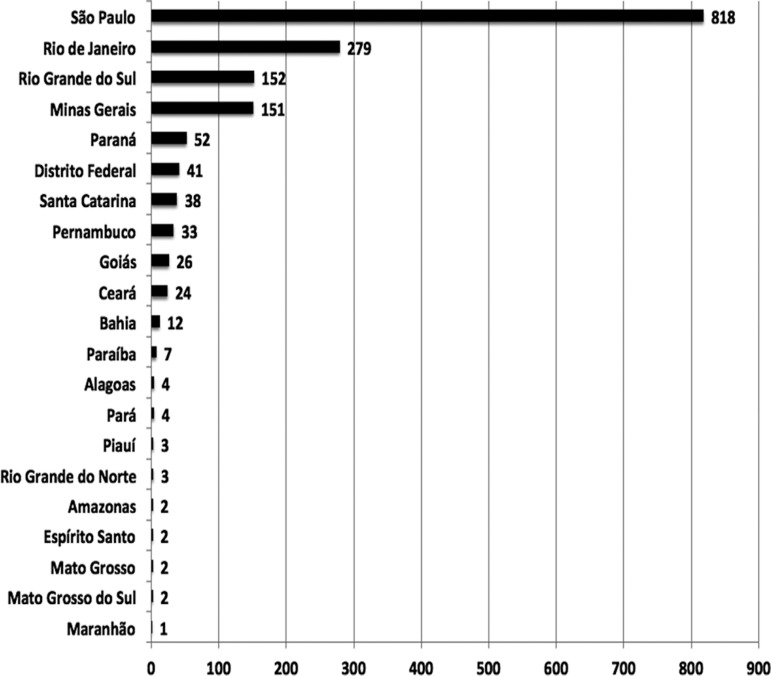
Number of publications according to Brazilian states.

The majority of institutions involved in publications were public universities, featuring São Paulo University (USP), which was responsible for 29% of results (Figure 3). Concerning distribution throughout Brazilian states, institutions located in the southeast and south regions had a significant participation, representing 75% and 14% respectively (Figure 4).

**Figure 5:**
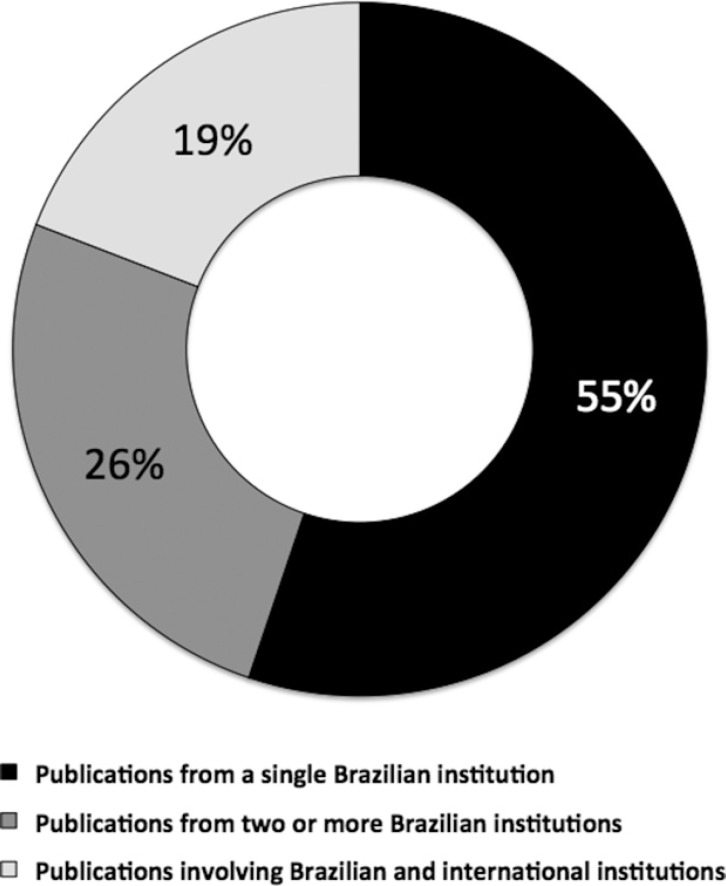
Distribution of collaborative and non-collaborative publications.

Fifty-five percent of publications involved a single Brazilian institution. Twenty-six percent involved two or more Brazilian institutions. Nineteen percent were collaborative publications involving Brazilian and international institutions (Figure 5). Distribution of publications according to the journal was divided almost equally between Brazilian and international periodicals, with a slight predominance of international journals (53% vs. 47%).

Rankings (Top 10) of the most used periodicals for publication are presented in Table 3 and Table 4, in addition to their representativeness amongst publications. When the analysis is focused on these periodicals, Arquivos de Neuro-Psiquiatria and Dementia & Neuropsychologia rank as the most important Brazilian journals. More than 11% and 6% of papers were published in these journals, respectively, over the period. In the same fashion, International Psychogeriatrics ranks as the most representative international periodical, accounting for more than 2% of the analyzed publications.

**Table 3:** Distribution of publications in international periodicals (Top 10).

International periodicals	Impact Factor*	N	%
International Psychogeriatrics	1.892	41	2.47
Journal of Alzheimer’s Disease	3.612	25	1.51
International Journal of Geriatric Psychiatry	3.086	18	1.09
Dementia and Geriatric Cognitive Disorders	2.812	18	1.09
Alzheimer Disease and Associated Disorders	2.688	14	0.84
Neurology	8.303	11	0.66
PLoS One	3.534	11	0.66
Journal of Neural Transmission	2.871	10	0.60
Journal of Neurology, Neurosurgery and Psychiatry	5.580	9	0.54
Journal of Biological Chemistry	4.600	9	0.54
Journal of the American Geriatrics Society	4.216	9	0.54

* Impact factor (2013) calculated by Thomson Reuters (Journal Citation Reports^®^)

**Table 4:** Distribution of publications in Brazilian periodicals (Top 10).

Brazilian periodicals	Impact Factor	N	%
Arquivos de Neuro-Psiquiatria	1.006*	188	11.35
Dementia & Neuropsychologia	0.247**	108	6.52
Revista de Psiquiatria Clínica	0.886*	68	4.10
Revista Brasileira de Psiquiatria	1.638*	50	3.02
Revista Brasileira de Neurologia	NA	25	1.51
Jornal Brasileiro de Psiquiatria	0.427**	24	1.45
Revista Brasileira de Medicina	0.059**	23	1.39
Clinics	1.422*	16	0.97
Brazilian Journal of Medical and Biological Research	1.034*	14	0.84
Revista Neurociências	0.135**	14	0.84

NA: not available,

* Impact factor (2013) calculated by Thomson Reuters (Journal Citation Reports^®^),

** Impact factor (2013) calculated by Scopus (SCImago Journal & Country Rank).

In relation to distribution by type of publication, most were original articles focusing on clinical research or literature reviews (Table 5).

**Table 5:** Distribution of types of publication.

Type of publication	N	%
Original articles – Clinical	600	36.2
Reviews	500	30.2
Original articles – Basic science	226	13.6
Case reports	86	5.2
Caregiver issues	76	4.6
Original articles – Human genetics	50	3.0
Letter	44	2.7
Original articles – Epidemiology	31	1.9
Editorial	26	1.6
Original articles – Pathology	10	0.6
Not classified	8	0.5
Total	1657	100%

## DISCUSSION

Brazil is the South American country with the highest scientific output in Medicine and the number of publications has increased substantially in the last two decades, following the same trend of world scientific output over this period.^9^

According to the present review, the overwhelming majority of institutions involved in scientific output on dementia and related disorders were public universities. Moreover, prominent institutions were located mainly in the southeast and south regions, although distribution of researchers throughout Brazilian territory was highly heterogenic, reflecting intrinsic variations within the country.

Our survey shows that output of Brazilian researchers in the area of cognitive disorders increased 4.98-fold from 2003 to 2013 (ten years) and 11.38-fold in the fifteen-year period of analysis (1999-2013). By comparison, in the last 20 years, from 1995 to 2014, the overall scientific output in Brazil increased by a factor of five.^10^

Despite this marked growth, the number of Brazilian scientific publications is still low in comparison to international standards.^11^ This is also true in the area of cognitive impairment and dementia, indicating that efforts are required to improve the output of Brazilian institutions. One possible strategy to accomplish this increase could be to encourage residents to participate in publications of scientific papers during their residence program.^12^

Forty-five percent of publications were collaborative, illustrating the power and importance of research networks converging toward a common interest. Thus, collaboration between different Brazilian universities and research institutions must be pursued by investigators and stimulated by funding agencies. To this end, specific grants aimed at supporting projects in cooperation in the area of cognitive disorders could be part of a national research plan by the federal government in conjunction with state funding agencies. The recent efforts of the Brazilian scientific community in the area of clinical investigation to create a local project within the Alzheimer's Disease Neuroimaging Initiative (ADNI) is also another strategy that might enhance the quality of research in the field.

In conclusion, both quantity and quality of Brazilian publications in international and Brazilian periodicals show a marked evolution in the last 15 years and we hope that this rising trend continues progressively in order to benefit patients not only in Brazil, but also worldwide.
